# Portrait of a cancer: mutational signature analyses for cancer diagnostics

**DOI:** 10.1186/s12885-019-5677-2

**Published:** 2019-05-15

**Authors:** Arne Van Hoeck, Niels H. Tjoonk, Ruben van Boxtel, Edwin Cuppen

**Affiliations:** 1 Center for Molecular Medicine and Oncode Institute, University Medical Centre Utrecht, Heidelberglaan 100, 3584CX Utrecht, The Netherlands; 2 Princess Máxima Center for Pediatric Oncology and Oncode Institute, Heidelberglaan 25, 3584CS Utrecht, The Netherlands; 3 Hartwig Medical Foundation, Science Park 408, 1098XH Amsterdam, The Netherlands

**Keywords:** Mutational signature, Cancer diagnosis, Cancer biomarkers, Cancer genomics, Molecular medicine, Whole genome sequencing

## Abstract

**Background:**

In the past decade, systematic and comprehensive analyses of cancer genomes have identified cancer driver genes and revealed unprecedented insight into the molecular mechanisms underlying the initiation and progression of cancer. These studies illustrate that although every cancer has a unique genetic make-up, there are only a limited number of mechanisms that shape the mutational landscapes of cancer genomes, as reflected by characteristic computationally-derived mutational signatures. Importantly, the molecular mechanisms underlying specific signatures can now be dissected and coupled to treatment strategies. Systematic characterization of mutational signatures in a cancer patient’s genome may thus be a promising new tool for molecular tumor diagnosis and classification.

**Results:**

In this review, we describe the status of mutational signature analysis in cancer genomes and discuss the opportunities and relevance, as well as future challenges, for further implementation of mutational signatures in clinical tumor diagnostics and therapy guidance.

**Conclusions:**

Scientific studies have illustrated the potential of mutational signature analysis in cancer research. As such, we believe that the implementation of mutational signature analysis within the diagnostic workflow will improve cancer diagnosis in the future.

## Background

Historically, cancer diagnostic and treatment decisions were predominantly based on tumor morphology, clinical symptoms, and the cancer site of origin. In the past decade, systematic analyses of cancer genomes have changed this paradigm [1], and the term ‘cancer’ now encompasses more than a hundred different diseases differentiated on the basis of varying combinations of cancer gene mutations [2, 3]. This development, together with the emergence of molecularly targeted drugs, resulted in an increase in molecular testing to support decision making in cancer diagnostics and treatment.

Thus far, the development of cancer diagnostics has mainly focused on identifying driver mutations that provide growth advantages to cancer cells and thereby promote tumorigenesis [2]. Genetic testing for driver genes can identify the biological characteristics of tumors. These genes can also act as direct targets for effective treatment. The rapidly growing number of drugs directly targeting proteins encoded by mutated driver genes has fueled the development of assays for the accurate detection of mutations for cancer diagnosis [4].

Although this knowledge has contributed significantly to drug development and improved cancer care, a substantial portion of patients do not benefit from this strategy because of poor response rates to targeted drugs and a lack of adequate biomarkers. Therefore, cancer diagnostics require better molecular characterization of tumors, as well as reliable biomarkers for patient stratification. Next-generation sequencing (NGS) technologies have emerged as an important tool to fulfill this unmet need. The capacity of NGS to analyze large panels of genes, up to complete cancer genomes, has enabled the generation of comprehensive catalogues of somatic mutations in cancer patients [5–7]. However, only a very small fraction of the identified variants are tumor drivers or actionable biomarkers. The vast majority of somatic mutations in a cancer genome are passenger mutations, which are not believed to be involved in cancer development. Nevertheless, it has recently been shown that these alterations can be used to provide insight into the history of the tumor and identify mutational processes that have occurred before and during tumorigenesis [8]. Somatic mutations can originate from exogenous factors, such as environmental carcinogens or UV radiation, or endogenous processes, such as normal mutational decay due to spontaneous deamination of methylated nucleotides, base misincorporation by error-prone polymerases, and unrepaired or incorrectly repaired DNA damage due to impaired DNA damage response (DDR) gene function (reviewed by Helleday et al. [9]). Interestingly, each of these leave a characteristic pattern of mutations, which have been dubbed ‘mutational signatures’ [8]. For instance, cells defective in homologous recombination repair (HRR) machinery or non-dividing cells must rely on alternatives to repair DNA breaks, such as non-homologous end-joining and alternative end-joining to repair double-stranded DNA breaks [10]. These repair processes are not error free and leave a characteristic mutational pattern, which has been shown to be useful for the identification of tumors deficient in HRR [11, 12]. Mutational signatures can therefore reflect the presence or absence of cellular processes in cancer cells. Because multiple endogenous or exogenous mutational forces can operate simultaneously or successively on the genome during a cell’s life span, the mutational catalogue of a cancer genome harbors a mixture of signatures shaped by different mutational processes. Some of these mutational processes are active continuously throughout the lifetime of the cancer cell (clock signatures) [13], whereas others are active periodically, some of which are influenced by the patient’s lifestyle [14].

It has recently been shown that mutational signatures can be biomarkers for specific characteristics of a cancer [8, 15]. As such, they bear potential clinical value as predictors of the therapy response in cancer [11]. An important prerequisite for mutational signature analysis is the availability of genome-wide mutational data across many independent cancers. As the cost of whole-genome sequencing decreases and the amount of available cancer mutation data grows, it is timely to consider mutational signature analysis a novel opportunity for biomarker discovery, tumor diagnostics, and treatment guidance.

### Signatures reveal mutation etiology

The first mutational signatures introduced were base substitutions. For these mutation types, a signature is characterized by the specific base change and its direct 5′ and 3′ flanking base. Because there are six classes of base substitution and 16 possible sequence contexts, there are 96 distinguishable trinucleotide changes. Therefore, mutational signatures can be distilled from large cohorts of sequenced cancer patients by a computational framework that attempts to decompose distinguishable recurrent patterns from the cohort’s 96-mutation matrix. Ultimately, each pattern represents the relative proportion of each trinucleotide mutation, which reflects a mutational signature. More theoretical details about the framework can be found in Alexandrov et al. [16], and Serena et al. [17] provides a chronological overview on mutational signature analysis in cancer.

Although mutational signatures are a relatively recent concept in cancer biology, the idea of linking mutational processes with mutational patterns is not new. The first studies linking specific mutation characteristics to various environmental mutagens, such as UV-radiation [18], smoking [19], and aristolochic acid [20], were focused on single cancer genes that were recurrently mutated in a wide range of cancers, such as *TP53* and *BRAF*. These studies provided the first evidence that mutational processes can leave characteristic patterns in the DNA that are visible and analyzable in tumor samples via the detection of distinct signatures [21]. In 2013, Stratton and his team introduced a computational framework that used nonnegative matrix factorization (NMF) to recognize multiple base substitution patterns in human cancers [15, 22]. Moreover, some of these patterns correlated with known mutagenic processes, indicating that this mathematical concept can extract biologically relevant information to unravel mechanisms underlying tumorigenesis [16]. Since this seminal study by Stratton’s group, the field of mutational signature analysis has grown rapidly in cancer biology. Currently, there are 30 different reference signatures described in primary cancer that are categorized in the COSMIC database (http://cancer.sanger.ac.uk/cosmic/signatures) [22]. However, additional signatures continue to be identified by various research groups [23–26], and methods to characterize cancer genomes in a similar way based on indels, structural variants, and copy-number changes are currently under development [27].

Comparing these signatures with the scientific literature, as well as statistically associating them with patient phenotypes, provided the first mechanistic insights into the etiology of a number of mutational processes. Mechanisms underpinning mutational signatures have been suggested for roughly half of the 30 COSMIC signatures. The establishment of large pan-cancer genomic datasets, such as The Cancer Genome Atlas (TCGA) [28], Welcome Trust Sanger Institute’s Cancer Genome Project [29] and the International Cancer Genome Consortium (ICGC) [1], were vital for these analyses. By doing so, exogenous processes (e.g., tobacco smoking and UV-exposure) and endogenous processes (e.g., APOBEC overactivity, deficiency in double strand break repair, and polymerase slippage) could be attributed to specific signatures. However, obtaining evidence that the proposed etiology of a signatures is a specific mutational process, based solely on data derived from cancer patients, is not straightforward. It is complicated by the lack of complete catalogues of true pathogenic driver variants and missing information on the environmental exposure history of the patient cohort. Additional complexities can be found in the heterogeneous landscape of mutational processes that is typically identified in individual cancers. Furthermore, the detected somatic mutations are the result of a balance between mutation-inducing and DNA repair processes, which are not fully independent, and mechanisms may vary between tissues. Therefore, more controlled experimental approaches are needed to determine the origin of a signature. We recently showed that the application of CRISPR-Cas9 technology in human colon organoids to delete key genes involved in specific DNA repair pathways, followed by genome-wide characterization of the resulting mutation patterns, is a powerful approach because it can link the observed signatures of the accumulated mutations directly to the biological functionality of the inactivated gene [30].

### Diagnostic mutational signatures

**Fig. 1:**
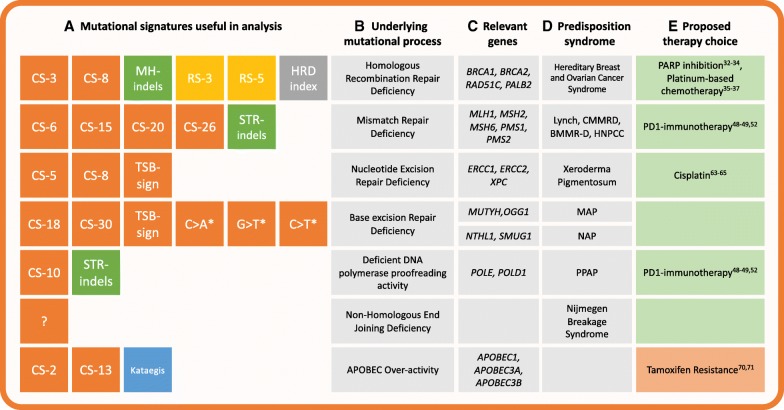
Mutational processes linked to treatment selection via mutational signatures. Mutational signatures in tumor genomes can reflect the activity of specific mutational processes and thereby provide support for therapy selection. Different types of mutational signatures (**a**) can be considered: base substitution signatures (orange), indel signatures (green), rearrangement signatures (yellow), geographically localized mutational phenomena (blue) or other signatures characterized by copy-number variations (grey). Diagnostic interpretation of characteristic signatures can contribute to therapy choice (**e**) and include (green) or exclude (red) patients from a treatment. Actionable pathways that can be identified by mutational signatures (**a**) mainly include DNA repair defects (**b**), which was confirmed by the presence of pathogenic mutations in the indicated genes in these pathways (**c**). The prevalence of germline pathogenic mutations in these genes is typically linked to a cancer predisposition syndrome (**d**). *Abbreviations: CS-[number], COSMIC signature; RS-[number], rearrangement signature; MH-indels, indels at microhomologies; STR-indels, short tandem repeat-mediated indels; TSB sigs, signatures showing transcriptional strand bias. APOBEC, apolipoprotein B DNA-editing complex; MAP, MUTYH-associated polyposis; NAP, NTHL1-associated polyposis; PARP, poly(ADP-ribose) polymerase; PPAP, polymerase proofreading associated polyposis.* * defects in base excision repair have been associated with these characteristic substitutions

Currently, the most notable advances in mutational signature analysis-based diagnosis are in the field of breast cancer. Tumors with mutations in *BRCA1/2* are defective in the HRR process. These tumors show promising responses to treatment with a PARP inhibitor (olaparib), a drug that decreases the DDR in cancer cells to a fatally low level [31–33]. DNA-damaging agents that directly induce double strand breaks, such as chemotherapy based on platinum salts, prove therapeutically efficient in these cancers as well [34–36]. Recently, a model that can accurately predict HRR deficiency (HRDetect) was developed for breast cancers [11]. This computational tool uses HRR-deficiency features from the complete mutation catalogue of base substitutions, indels, and structural rearrangements. The use of this tool revealed that microhomology-mediated indels, two COSMIC signatures (further referred to as CS) and two rearrangement signatures (further referred to as RS) correlated with HRR deficiency (Fig. 1). By accounting for their mutational contribution, HRDetect could predict BRCAness (i.e., a *BRCA1/2*-associated phenotype) with a sensitivity of almost 100%, which is an improvement on the sensitivity obtained by more traditional copy number based tests (~ 60%) [37] and functional assays of HRR deficiency (~ 80%) [38]. HRDetect identified 44 cancers that carried a germline or a somatic *BRCA1/2* variant in a cohort of 560 breast cancer patients and, interestingly, in 47 cancers demonstrating BRCAness in which no pathogenic variant in *BRCA1/2* was detected. The latter category can possibly be explained by the epigenetic inactivation of *BRCA1/2* or the inactivation of other components involved in HRR.

The HRDetect tool demonstrates that signature analysis can be deployed to successfully identify BRCAness in patients without the need for prior knowledge of *BRCA* mutations. Polak et al. [12] found similar results in a different breast cancer cohort, and pointed out that cancers carrying a somatic event in *BRCA1* (*n* = 36, cohort size = 995) or *BRCA2* (*n* = 34, 995) had a stronger contribution from CS-3. Interestingly, cancers that showed epigenetic silencing of *BRCA1* (*n* = 32, 995) or *RAD51C* (*n* = 23, 995), or that carried germline *PALB2* (n = 3, 995) or *RAD51C* (*n* = 1, 995) mutations, also displayed an increased contribution from CS-3. Because epigenetic modifications cannot be directly verified by traditional diagnostic methods, identifying mutational signatures associated with HRR defects can increase the number of patients who would benefit from treatment with PARPi and platinum-based drugs [11]. Recently, we validated this strategy in breast cancer organoids by subjecting organoids derived from a patient who displayed a high contribution from CS-3 mutations to two different PARPi drugs [39]. These organoids were sensitive to PARPi, whereas breast cancer organoids negative for CS-3 did not show any response, illustrating the principle that CS-3 can act as a useful marker for PARPi sensitivity in cancer. A recent retrospective study computed HRDetect scores for 93 advanced breast cancer patients, 33 of which were treated with platinum chemotherapy [34]. All patients scoring high for HRR deficiency showed a significantly association with clinical improvement on platinum-based therapy. These findings provide evidence for the use of mutational signatures as sensitive biomarkers for HRR defects, and can inspire the design of therapeutic trials.

Moreover, mutational signature analysis to detect HRR deficiency could be applied to many different cancer types beyond breast cancer. Germline mutations in *BRCA1/2* have long been known to affect the risk of ovarian cancer [40] and pancreatic cancer [41]. Biomarkers for HRR deficiency were found in 24 additional cancer classes or cancer-associated syndromes [8, 42–44]. These findings suggest that HRR deficiency and the associated therapeutic benefits may apply to a greater number of patients than is currently appreciated. Indeed, in a study on pancreatic cancer, all patients that responded to platinum-based chemotherapy harbored the BRCA-associated CS-3 [45]. These examples indicate that an effective response to specific anti-cancer drugs is more dependent on specific functional defects in a tumor than by the organ in which this tumor is located. Nevertheless, the efficacy of HRDetect in selecting patients of all cancer types for PARPi, platinum-based, and/or immune-based therapy needs testing in (pre)clinical trials.

Similar tactics could be employed for other mutational process signatures as well. DNA mismatch repair (MMR) corrects stochastic errors by polymerases that arise during DNA replication [46]. A deficiency in MMR and DNA proofreading results in increased mutational load of base substitutions and instability at tandem repeats of short nucleotide sequences (a feature called microsatellite instability [MSI]) [47] (Fig. 1). Colorectal cancers with MMR deficiency are sensitive to pembrolizumab [48] and nivolumab [49], which are both inhibitors of the programmed death 1 (PD1) immune checkpoint. In 2015, the Consensus Molecular Subtypes (CMS) Consortium subcategorized all hypermutated MSI cancers in one CMS group (CMS1, 14% of colorectal cancers) based on gene expression data. Mutational signature analysis demonstrated that MSI colorectal cancers leave specific mutational signatures (CS-6, CS-15, CS-10, CS-20, and CS-26) [8, 9], which can be used to identify MMR deficiency in cancers [26, 30]. Recently, we validated the association between MMR deficiency and a CS-20-like signature in colon organoids that lack the essential MMR gene *MLH1* [30]. These organoids were exclusively characterized by this base substitution signature accompanied by small indels (< 3 bp) within a tandem repeat context (Fig. 1). These mutation characteristics could be used to identify colon cancer patients with MMR deficiency even when that deficiency is caused by epigenetic mechanisms such as the well-studied *MLH1* promotor methylation. Although MRR-deficient cancers dominate in colorectal cancers [50], signature analysis revealed MMR-deficient pancreatic cancer as well (*n* = 3, 180) [51]. Thus, as in the case of HRR deficiency, signature analysis might be a convenient approach to simultaneously screen for MMR deficiency to identify patients who would benefit from immunotherapy, regardless of the cancer’s tissue of origin [52]. Indeed, in a follow up study, Le et al. showed that PD1 inhibition is not just successful in treating colon cancer with MSI but also in treating 11 other cancer types with MMR-deficiency [53].

Base excision repair (BER) is a third category of DNA repair that could potentially be discerned by mutational signatures. Defects in BER components *SMUG1*, *OGG1*, and *NTHL1* result in higher rates of C > A transversions (*SMUG1* and *OGG1*) [54, 55] and C > T transitions (*SMUG1* and *NTHL1*) [56, 57]. These findings indicate that the failure of BER processes might also leave specific predictive marks. Indeed, using CRISPR/Cas9-mediated knockout of *NTHL1* in colon organoids, we have shown that *NTHL1* deficiency results in increased mutations, which can be attributed to CS-30 [30]. This signature had been identified in only a single cancer patient within a breast cancer cohort [22]. Upon examining the germline of this patient, we identified a heterozygous mutation causing a premature stop codon in *NTHL1*, with loss of heterozygosity in the tumor. Mutations in *MUTYH*, a BER- and nucleotide excision repair (NER)-associated protein, are specifically associated with CS-18 [58] and a CS-18-like signature [26, 59]. Because BER and NER can both be coupled to transcription [60, 61], more specific mutational signatures could possibly be dissected when such genomic features are taken into account (including CS-4, CS-5, CS-8, CS-12, CS-16, and CS-22 – see Fig. 1) [8]. For example, a specific mutational signature that closely resembles CS-5 has been associated with defects in *ERCC2*, a core protein of the NER pathway [62]. Importantly, this signature was significantly increased in responders to cisplatin compared to non-responders, and other studies have also confirmed a positive response to cisplatin in NER-deficient patients [63–65]. However, the studies of CS-5 also illustrate one of the limitations of the use of mutational signatures. It is now considered that this siganture represents a universal ageing signature, as does CS-1 [13, 30], since both signatures have been observed in healthy cells. CS-5 therefore has little diagnostic value, but it remains to be shown whether quantitative analyses reveal a robust association of NER deficiency with increased levels of CS-5 mutations. Furthermore, not all NER-deficient tumors show the same signature contribution, suggesting that distinct mutational processes related to NER deficiency might be active. Indeed, recent findings from our laboratory indicate that deficiency in global genome NER results in a tissue-specific increase in mutations, which can be attributed to CS-8 [66].

In addition to DNA repair deficiencies, other cellular processes can leave informative signatures in tumors. Activation of the RNA-editing enzyme APOBEC constitutes part of the cellular immune response to viruses and retrotransposons, but overactivity of APOBEC is a driving force of somatic hypermutation [67]. This implies that tumors with APOBEC overactivity could be treated by lethal mutagenesis, which consists of administration of drugs stimulating mutation rates past a lethal threshold, thereby stimulating programmed cell death [68]. APOBEC enzymes have also been proposed to drive cancer evolution, heterogeneity, and therapy resistence [69]. APOBEC overactivity has been shown to promote drug resistance to the cancer drug Tamoxifen [70, 71], perhaps due to APOBEC-driven intratumor heterogeneity. The APOBEC-associated signatures CS-2 and CS-8, as well as an associated phenomenon of clustered mutagenesis called kataegis (Fig. 1), have been found in more than half of the investigated cancer types [6]. Additionally, later studies found these signatures in in a range of cancer types [24, 72–74] and directly linked them to an *APOBEC3A/3B* germline deletion allele in breast cancer [75]. Detection of APOBEC overactivity could therefore be useful in a wide range of cancer types. Moreover, mutational signature analysis allows discrimination between the signatures of different APOBEC-subtypes [76]; the APOBEC3B subtype could be further subdivided with clustered mutational signatures [77], which means even more specific targeting could be possible. For example, APOBEC stimulators might be used to stimulate lethal mutagenesis.

### Stratification of cancer patients

**Fig. 2:**
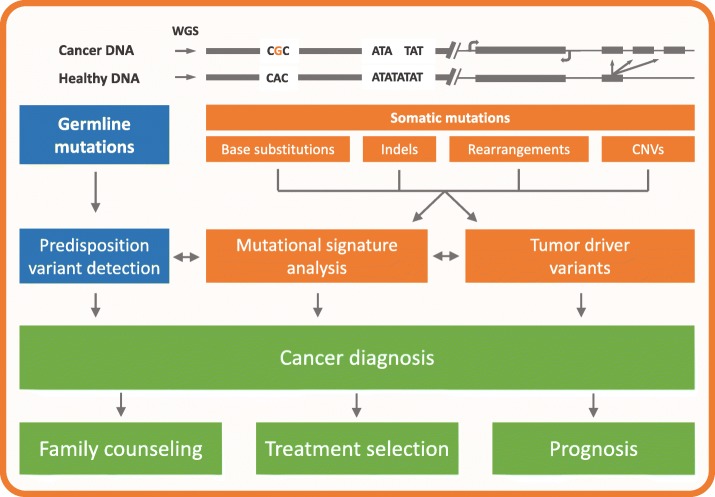
Mutational signature analysis as a tool in cancer diagnostics. A patient who is diagnosed with cancer will undergo biopsy of both the tumor tissue and a healthy tissue sample (e.g. blood). The entire DNA of both specimens will then be read using whole-genome sequencing (WGS), which allows the characterization of somatic mutations in the form of base substitutions, indels, rearrangements, copy-number variations (CNVs) and variations thereof. The healthy sample can be used to characterize predisposition variants, and somatic events can identify potentially actionable somatic tumor driver variants. Mutational signature analysis can provide additional evidence to support the interpretation of these measurements, such as for the interpretation of Variants of Unknown Significance (horizontal arrows), but can also provide direct support for the cancer diagnosis. The result of this workflow will influence clinical interventions such as treatment decisions and family counseling if a predisposition variant has been identified, and allows for stratification of patients towards effective anti-cancer drugs (precision medicine) to improve the patient’s outcome (prognosis)

In addition to using mutational signatures as a genomic biomarker for targeted therapeutics, mutational signature analysis presents possibilities in the stratification of patients (Fig. 2). For instance, breast cancer is among the most common types of cancer worldwide, with an estimated incidence of 1.7 million cases in 2012 [78]. Around 5–10% of all breast cancers are attributed to somatic or germline mutations in the genes *BRCA1* and *BRCA2* [79]. However, HRR deficiency is currently not an intrinsic subclass in breast cancer diagnostics, although this cohort may have a better prognosis when treated with specific drugs. A few recent studies have applied mutational signature analysis to identify which patients are most likely to respond to certain therapies, including studies of patients with esophageal adenocarcinoma (EAC) [43], pancreatic ductal adenocarcinoma (PDAC) [44], oral squamous cell carcinoma (OSCC) [80], gastric cancer [25], and prostate cancer [81].

EAC is an illustrative example. Highly heterogeneous mutational landscapes and a current lack of efficient stratification methods has led to the generally poor performance of targeted therapeutic approaches [43, 82]. However, in a cohort of 129 EAC patients, Secrier et al. [43] were able to define each patient’s tumor by its dominant mutational signature and performed hierarchical clustering to stratify tumors into three subgroups with distinct etiologies. The first subgroup exhibited faults in the HRR pathway and was characterized by CS-3, and could therefore benefit from PARP inhibitors or platinum-based chemotherapy. The largest subgroup predominantly showed CS-17, a signature that does not yet have a defined etiology but could be related to gastroesophageal reflux [83]. In this subgroup of cancers, an increased response to WEE1/CHK1 inhibitors was observed. In addition, CS-17 has been shown to correlate with high neoantigen loads, which could implicate these patients for immunotherapy [84–86]. The final subgroup predominantly showed the signatures CS-1, which is age-related, and CS-18, which has no consensus etiology as yet but has been suggested to be associated with damage from reactive oxygen species (ROS) [26, 59]. Although Secrier et al. suggested traditional chemotherapy for these patients, the clinical meaningfulness of this subgroup is questionable, because there is no other obvious treatment alternative for these patients at this time. Uncovering the mechanisms underlying CS-18 and CS-1 will possibly energize the search for therapeutic potential in this subgroup. Molecular stratification of cancer patients based on mutational signatures is used in a growing number of studies, although in variable forms. Whereas EACs and gastric cancers were classified using predominantly mutational signature analysis [25, 43], PDACs and OSCCs were stratified using mutational signatures as part of an integrated genomics approach [44, 80]. However, other tumor characteristics must often contribute to a comprehensive tumor diagnosis and treatment decision, because not all therapies are directly related to the mutational processes driving cancer. Nevertheless, mutational signatures already provide relevant information for treatment selection in at least some subgroups. In addition, evaluation of mutational signatures is an interesting approach that could be explored for the stratification of patients in clinical trials.

### Revealing cancer predisposition

The majority of cancers are believed to result from somatic mutations [2]. Nevertheless, up to 10% of the cases can be attributed to inherited variants present in the patient’s germline [87]. Exome sequencing studies in the last decade have revealed many new predisposition gene candidates, and whole-genome sequencing (WGS) pan-cancer studies will likely unravel new predisposition genes in the future, such as non-coding driver variants [88]. Mutational signature analysis could potentially be applied as a powerful screening tool to uncover new pathogenic inherited mutations affecting mutation accumulation, and as a validation method to accurately classify variants of uncertain significance (VUS) as either pathogenic or benign (Fig. 2). For instance, we have identified a germline *NTHL1* variant in a breast cancer patient by screening for CS-30 conribution [30]. Polak et al. revealed that nearly all samples showing a pathogenic *BRCA1/2* germline variant and loss of the intact allele were positive for CS-3 in the TCGA breast cancer cohort, and accurately classified 12 *BRCA1/2* VUSs [12]. The integration of indel and rearrangement signatures can even segregate *BRCA1* deficient tumors from *BRCA2* mutants [11]. It is worth mentioning that not all heritable breast cancers harbor germline variants solely in *BRCA1/2*, indicating that predisposing variants in other genes likely exist and contribute to hereditary breast cancer via altered mutation accumulation.

A more advanced approach could incorporate the evolutionary dynamics of the signatures to identify early-onset signatures which, together with true driver detection, can be used to trace predisposition variants from tumor-only sequencing data [89]. Such an approach has been tested in 15 ultrahypermutated cancer patients (> 100 mutations per Mb) and in each individual, a germline MMR mutation was found. Moreover, this analysis was performed on panel sequencing data, which covers a sufficient number of nucleotides to identify early-onset signatures from highly mutated cancer types but is likely not adequate for less mutated cancer types. However, this strategy can be implemented in a whole-genome/−exome framework to predict predisposition variants in other cancer types.

The application of mutational signature analysis to reveal cancer predisposition could be an important step forward in familial cancer diagnosis. For instance, many colorectal cancer patients harbor mutated predisposition genes that can be classified into distinct colorectal cancer subtypes including polymerase proofreading associated polyposis (PPAP), *MUTYH*-associated polyposis (MAP), *NTHL1*-associated polyposis (NAP), and Lynch syndrome. These subtypes are pathologically very similar and therefore difficult to identify, requiring extensive multifactorial testing [90, 91]. However, PPAP has been associated with a distinct mutational signature, CS-10 [8]; MAP with two signatures, one CS-18-like [58] and a similar signature currently named signature 36 [26]; and NAP with CS-30 [30, 57]. The clinical value of detecting these predispositions is shown in Fig. 1. In addition, Lynch syndrome can be identified using the MMR-associated signature CS-6 [8] and indel signatures [30]. Indeed, a study aiming to detect Lynch syndrome used the aforementioned two Lynch syndrome-associated signatures and the PPAP-associated signature CS-10 to distinguish these two groups of patients [89]. However, for most of these syndromes, more research is required to validate the signatures. Additional studies of larger, selected cohorts can help unravel which syndromes are linked to which signatures. In addition, it is important to study whether other predisposition syndromes, not functionally linked with DNA repair deficiency, can be associated with a specific mutational pattern. These studies might best focus on hereditary cancer syndromes that are currently difficult to identify with targeted gene panels, such as Cowden syndrome [92]. Furthermore, additional studies are necessary to evaluate the efficacy of mutational signature analysis in identifying different hereditary cancer types, particularly because different syndromes may converge on the same signature and be indistinguishable. Nevertheless, the assignment of germline mutations in cancer patients has several important clinical implications, because these variants can serve as sentinels for identifying families with high risk for cancer development. Family members carrying pathogenic germline variants could be encouraged to obtain genetic counseling, take preventive measures, or enter increased surveillance programs (Fig. 2).

### Identifying tumor tissue of origin

Roughly 3% of all new cancer cases are diagnosed as a cancer of unknown primary (CUP) [93]. Furthermore, substantial uncertainty about the tissue of origin remains, especially when the cancer is metastatic or poorly differentiated; this complicates treatment because most targeted drugs are tumor type-specific. Mutational sequencing data could support histopathological examination in identifying the cancer site of origin. Comprehensive mutational signature analyses have shown that tumor types leave distinctive patterns of somatic mutations. For example, CS-12 and CS-16 are so far exclusively associated with liver cancer [94], and ovarian cancer typically harbors a high number of structural variants [95]. Such tissue-specific patterns, or a combination thereof, could be exploited to accurately decipher the primary tissue type. The ICOMS [96] (inferring cancer origins from mutation spectra) tool and TumorTracer [97] are two examples of well-trained classifiers that utilize TCGA and COSMIC data to infer the origin of distinct primary tumor sites. Although these tools deliver performance scores that may be accurate enough to aid in the clinical diagnosis of CUPs, the use of pan-cancer WGS data and advanced signature extraction methods will likely lead to more accurate approaches [98].

### Existing challenges

Thus far, we have discussed the current state and potential diagnostic value of mutational signature analysis, as well as applications for the detection of germline predisposition mutations and the determination of organ of origin for CUPs. However, clinical integration of such detection requires critical examination and further refinement of these signatures, and some obvious weaknesses and limitations must be addressed. First, the current 30 COSMIC signatures are derived from a mix of whole-exome sequencing (WES) and WGS data (10,952 whole exomes and 1048 whole genomes). This has resulted in discrepancies between WES- and WGS-derived signatures; for example, certain processes specifically act on coding or non-coding elements, such as transcription-coupled repair. This heterogeneity could be removed by creating WES- and WGS-specific signatures. This should ideally rely on the most comprehensive inventories obtained by WGS, because this also maximizes the ability to obtain insight into the underlying biological mechanisms. For clinical use, however, refitting of predefined signatures on WES data is likely feasible and more cost-efficient, which would make mutational signature analysis more broadly applicable. Second, a number of the current signatures are identified in only a few genomes at low contributions [8]. Their relevance should be substantiated before they are used in refitting approaches, because such signatures may mask the contributions of other signatures due to overlapping features. Likewise, signatures observed in single cohorts likely represent artifacts due to sequencing errors or from inadequate somatic mutation calling pipelines [99]. Consequently, the identification of such artifactual signatures makes it valid to question and optimize the sensitivity and specificity of the mutation calling strategy. Alternatively, artifactual signatures can be included to capture predefined false positive mutations as for example in single cell sequencing that generates numerous T > C mutations [100]. Third, not all mutational signatures will lead to targeting approaches or clinical advice. It is arguably unlikely that age-related CS-1, which is present in approximately 70% of all cancer types, can be translated to any form of prevention or treatment. Fourth, the accuracy of mutational signature extraction decreases when a multitude of mutational processes are or have been active in a sample, when low numbers of mutations are present (e.g. pediatric cancers and adult acute myeloid leukemia (AML) [101]), and when mutational signatures are relatively similar. Fifth, it is preferable to distinguish historical mutational processes from those that are presently ongoing to identify a signature-based treatment. For example, targeting APOBEC overactivity, a process that is known to operate transiently, solely on the presence of its signature will not necessarily affect patient survival. Likewise, subclones within tumors that have lost the activity of certain mutational processes will still contain their characteristic signatures within the genome. Moreover, subclones that have become the dominant clone during cancer recurrence after the first stages of treatment will still show their historic mutational signatures. In a diagnostic setting, mutational processes may be classified as historical or ongoing by analyzing samples from serial biopsies or biopsies from different sites within the tumor. Alternatively, active signatures can be characterized computationally by focusing on subclonal variants, because they are considered to originate from recent processes in local portions of the cancer. More sophisticated computational strategies also exist to assess the evolutionary history of mutational processes [102, 103].

It is important to mention that signatures of mutational classes other than base substitutions have enjoyed less attention. This is partly due to the known lower sensitivity and specificity of current algorithms used to call indel and structural variant mutations, which results in noisier data and more challenging extraction of biologically relevant signatures, as well as the higher complexity of defining other signatures [9]. The context of these signatures includes features beyond neighboring nucleotides, such as length, location, repeat engagement, copy-number changes, involvement of microhomology, and other biologically relevant attributes. Regarding indels, two distinct informative signatures were defined by Stratton and colleagues in breast cancer [9, 15]. The first indel signature is characterized by small indels (1–5 bp) flanked by short tandem repeats (STRs), and the second is characterized by larger indels (up to 50 bp) present in short stretches of identical sequences at the breakpoints (microhomology). Regarding structural rearrangements, six signatures (RS1-RS6) based on rearrangement type (duplications, deletions, translocations, inversions), degree of clustering, and size have been identified by analyzing 560 breast cancer genomes [22]. These indel and RS signatures are also found in liver cancer [94], highlighting the robustness of these preliminary indel and RS signatures. More signatures are likely to be recognized in the near future as techniques to identify indels and rearrangements develop and as cancer genomes are more systematically analyzed [104]. Additional relevant parameters may be incorporated into signatures in the future as well, including genomic features such as transcriptional strand bias [8, 105], replication timing [106, 107], genomic position [77], chromatin organization [108, 109], and other relevant genomic features [110]. For instance, heterogeneity in mutation rate has been observed within a single gene that is associated with higher mismatch repair activity in exonic regions [111], and clustered mutation signatures are related to variable APOBEC activity and tobacco smoking [77]. The inclusion of such parameters into patterns increases the resolution of mutational signatures to distinguish different processes. These parameters could also be clinically meaningful as stand-alone signatures, such as indels in STRs to identify MMR deficiency [112]. Which parameters must be incorporated into signatures and which can stand alone is a question that could potentially be addressed by feature correlation analyses of very large cancer genomics datasets. Furthermore, incorporating biases into signatures enhances the power of mutational signature analysis to detect underlying mutational processes.

The algorithmic approach behind mutational signature analysis still requires further development. A recent study validated a number of peer-reviewed mutational signature frameworks and found large variation in signature exposures, of which NMF gave on average the largest decomposition error [113]. Furthermore, NMF relies upon large cohorts of cancer genomes to accurately extract signatures and cannot efficiently analyze samples with high mutational load. Hence, a growing number of bioinformatics studies are attempting to address the shortcomings of NMF [16] by proposing and testing different mathematical approaches for problems such as defining the optimal number of signatures in a sample [114–120]. Alternatively, after a complete set of mutational signatures has been verified, the contribution of these predefined signatures (e.g., those currently recorded in the COSMIC database) could be refitted on the genomic data of a single patient [117, 119]. The latter strategy might prove faster and more cost-effective [43] and, most importantly, is applicable at the single patient level, which is a requirement for use in a clinical diagnostic setup. Methods for refitting known signatures to mutation inventories are still in their infancy, and are faced with challenges due to the overlapping characteristics of signatures, making it difficult to assign individual mutations to specific signatures. Hence, additional specific genomic features (e.g. broader mutation context, strand biases, association with functional elements) exclusively linked to a signature might be crucial to accurately asses the contribution of highly similar signatures and could simultaneously make refitting approaches more accurate.

Also, not all forces driving tumorigenesis might be detectable by DNA mutation analyses. Epigenetic modifications are another important cancer driver mechanism, but such alterations are not detected by routine WGS. It has been suggested that epigenetic changes, as detected by other targeted or genome-wide techniques, could be integrated into mutational signature analysis if need be [11]; however, no framework has been published yet.

### Feasibility and costs

Despite the recent advances in DNA sequencing technology and the consequent wave of studies using mutational patterns, diagnostic application of mutational signatures is still at an early stage of development. Certain mutational signatures can be linked to mutational processes and, via this route, to a treatment plan. To date, however, studies on how mutational signature-based subtyping translates to treatment response are largely absent. Studies using HRDetect or stratifying studies on the basis of mutational signatures do demonstrate a correlation with therapy response [11, 43, 44], but these studies were performed retrospectively. Therefore, the major challenge for mutational signature analysis will be to predict treatment response in a prospective study.

In addition, mutational signature analysis requires NGS data to accurately identify somatic mutations, preferably from WGS data with sufficient sequencing depth, accompanied by a matched healthy sample. WGS-derived data contain 20–50 times more mutations than do data from whole exomes [121, 122]. Hence, the decomposition of a patient’s mutational profile into de novo signatures using WES data may generate unstable signatures, as discussed above. However, Polak et al. [12] successfully detected BRCAness in the TCGA WES-derived dataset using an optimal threshold of 37 CS-3 associated mutations (AUC = 0.82). Therefore, refitting on robust mutational signatures and optimizing threshold levels may well work with only exome sequencing data of the diagnostic sample [98, 100]. Regarding sequencing depth, only a small drop in sensitivity was observed in the WGS breast cancer analysis when data with a 30-fold read depth was down-sampled to a 10-fold read depth (r = 0.96), with a remaining sensitivity of 86% for low-coverage sequencing data [12]. Similarly, a simulated 10-fold read depth could be successfully used to identify the dominant signature for EAC-patient stratification [43], although the required read depths will also strongly depend on the percentage of tumor cells in the sample and the tumor heterogeneity. Furthermore, the current somatic calling pipeline demands a matched healthy DNA sequence to distinguish somatic mutations from germline variants. However, the establishment of comprehensive population resources and well-trained computer models could potentially overcome this requirement without losing the detection power of mutational signature analysis.

Studies presenting the feasibility of mutational signature analysis for cancer patients have mostly used high-quality DNA extracted from fresh-frozen biopts. However, in clinical practices, such specimens are routinely fixed in formalin and paraffin-embedded (FFPE) for histopathological diagnosis, which lowers DNA quality [123]. Nevertheless, HRDetect (using 30-fold read depth) sustained high probability using FFPE tissues, indicating that mutational signature analysis may work in the current framework of molecular pathology. However, low-exposure signatures such as CS-3 and CS-5 might be lost in FFPE-induced noise [11].

Overall, it is difficult to draw conclusions on the cost-effectiveness of mutational signature analysis at this moment, although it is clear that costs for WGS are still clearly prohibitive for routine application in most clinical studies. However, when the potential of WGS to replace the multifactorial testing of mutated genes and to allow better patient stratification is met, cost-effectiveness could likely be reached, because WGS costs are only a fraction of total clinical study costs or the costs of current novel targeted treatments. In addition, with the decreasing costs of NGS, full commitment to WGS might be less of an issue at some point in the future.

### Clinical trials

Mutational signature analyses have been applied in research, but not yet in the clinical setting. Currently, our theoretical understanding of the mechanisms by which mutational signatures accumulate is still relatively rudimentary. However, the findings that are gained by WGS analysis open up the question of when WGS analyses will enter routine clinical cancer care. Therefore, examination of the mutational landscapes in clinical trials exploring the accuracy of this approach in a wide range of cancer types are a pertinent next objective. So far, only a very limited number of clinical trials (as reported on clinicaltrials.gov) have been initiated to examine the clinical relevance of mutational signatures.

The potential therapeutic efficacy of the PARP inhibitor olaparib in *BRCA*-mutated tumors has been assessed in clinical trials in breast cancer (NCT00494234 – completed), ovarian cancer (NCT00494442 – completed; NCT00753545 – completed; NCT00679783 – completed), prostate and pancreatic cancer (NCT01078662 – completed, NCT02677038 – recruiting; NCT02184195 – recruiting) and has been approved by the FDA in 2014 [124]. Currently, other PARP inhibitors are being tested for BRCA-deficient cancers in clinical settings, such as veliparib (NCT01149083) and rucaparib (NCT02855944), and platinum-based chemotherapy has been tested in prostate cancer (NCT01289067). However, these patients were mostly screened using targeted assays for germline and somatic BRCA mutations. The development of a companion diagnostic biomarker that relies on signatures (such as HRDetect) could guide treatment of HRR-deficient cancer types beyond those carrying *BRCA* mutations in the cancer types discussed above, and thus increase the target population. In this context, one trial (NCT01042379) investigated a BRCA-signature from gene expression data that was developed within the EU FP7 RATHER project, which showed promise in predicting the response to PARP inhibitor veliparib in combination with carboplatin [125]. However, the prognostic and diagnostic value of BRCA-associated signatures from somatic mutations remains to be assessed through a prospective clinical trial, with participants being selected based on the mutational signatures of their tumor.

One trial (NCT02710396) is currently recruiting patients to explore the mutational smoking signature as a potential biomarker in advanced non-small cell lung cancer treated with pembrolizumab. This PD1-blocking agent was FDA-approved in May 2017 for cancer patients diagnosed with microsatellite instability-high (MSI-H) or mismatch repair deficient (dMMR) cancers. Currently, MSI detection depends on a small number of known microsatellite loci or mismatch repair genes, and has limited reliability [126]. However, NGS data can offer highly accurate detection of MSI [127, 128]. Pembrolizumab was the first FDA-approved cancer treatment solely based on a genetic biomarker, rather than in combination with a primary tumor type. This decision opens up the route for additional biomarkers that focus on genomic profiles. In this context, a clinical trial (NCT02750657) has been set up to study the potential of mutational signature analysis for better treatment selection in PDAC, which is currently recruiting patients and might prove important for realizing the diagnostic potential of mutational signature analysis [44].

## Conclusion

In conclusion, cancer diagnosis may benefit from the implementation of mutational signature analysis, which is complementary to existing diagnostic approaches such as analyses of driver mutations in oncogenes and tumor suppressors. The identification of HRR deficiency in breast cancer and other cancers suggest the potential for a broader application of mutational signature analysis in different cancer types. Moreover, the detection of additional signatures suggests that similar developments could occur in the diagnosis of a broader range of DNA repair defects. Mutational signatures are proving to be clinically useful biomarkers for a growing range of cancer types, and signatures have already been shown to be useful for prognosis in several studies, such as the prediction of responses to conventional chemotherapy, targeted therapy, and immunotherapy approaches. Moreover, mutational signatures are found to be powerful biomarkers for the identification of hereditary cancer syndromes, providing opportunities for cancer prevention, monitoring, and early detection strategies.

Despite these promising results, mutational signature analysis will need further research to define universal reference signatures based on all types of mutational events and relevant genomic features, as well as to delineate the underlying mutational processes. This will require analyses of extensive, and more diverse, cancer genome sequencing datasets, as well as the targeted manipulation or perturbation of experimental models. Moreover, it is important that prospective clinical trials are undertaken to assess the effectiveness and accuracy of mutational signature analyses in predicting response to therapy. Finally, for patients to benefit from these developments, transparency regarding technical advances in algorithms and sharing of methods and data are imperative for the timely and responsible transfer of mutational signature analyses from the research domain to the clinical setting.

## Abbreviations

AML: acute myeloid leukemia
BER: base excision repair
CMS: Consensus Molecular Subtypes
CS: COSMIC signatures
CUP: cancer of unknown primary
DDR: DNA damage response
EAC: esophageal adenocarcinoma
FFPE: formalin-fixed paraffin-embedded
HRR: homologous recombination repair
ICGC: International Genome Consortium
ICOMS: inferring cancer origins from mutation spectra
MAP: MUTYH-associated polyposis
MMR: DNA mismatch repair
MSI: microsatellite instability
NAP: NTHL1-associated polyposis
NER: nucleotide excision repair
NGS: next generation sequencing
NMF: nonnegative matrix factorization
OSCC: oral squamous cell carcinoma
PARPi: PARP inhibitors
PD1: programmed death 1
PDAC: pancreatic ductal adenocarcinoma
PPAP: polymerase proofreading associated polyposis
ROS: reactive oxygen species
RS: rearrangement signatures
STR: short tandem repeat
TCGA: The Cancer Genome Atlas
VUS: variants of uncertain significance
WES: whole-exome sequencing
WGS: whole-genome sequencing
